# Supplementary material for: Serum Response Factor (SRF) Drives the Transcriptional Upregulation of the MDM4 Oncogene in HCC
Source: Cancers (Basel). 2021 Jan 8;13(2):199. doi: 10.3390/cancers13020199 (PMC7829828; doi:10.3390/cancers13020199)
Supplement: Supplementary file 1 [file cancers-13-00199-s001.pdf]

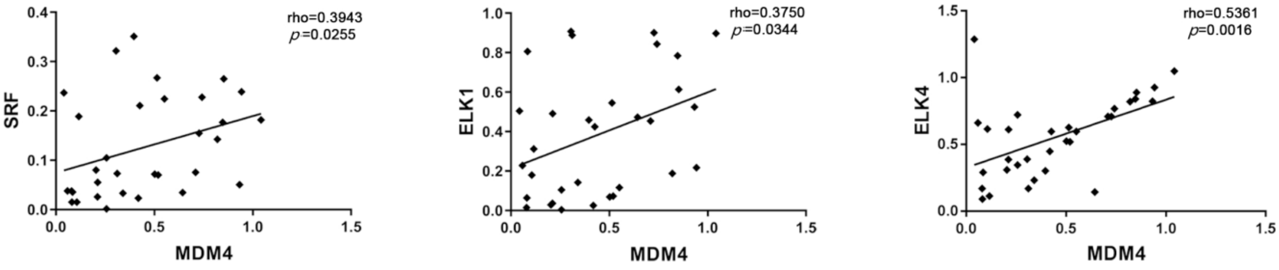

Figure S1. *MDM4* correlates with *SRF*, *ELK1* and *ELK4* at expression levels. A positive association was detected between *MDM4* and *SRF*, *ELK1*, *ELK4* mRNA levels in human HCC specimens of a second cohort ($n = 32$).

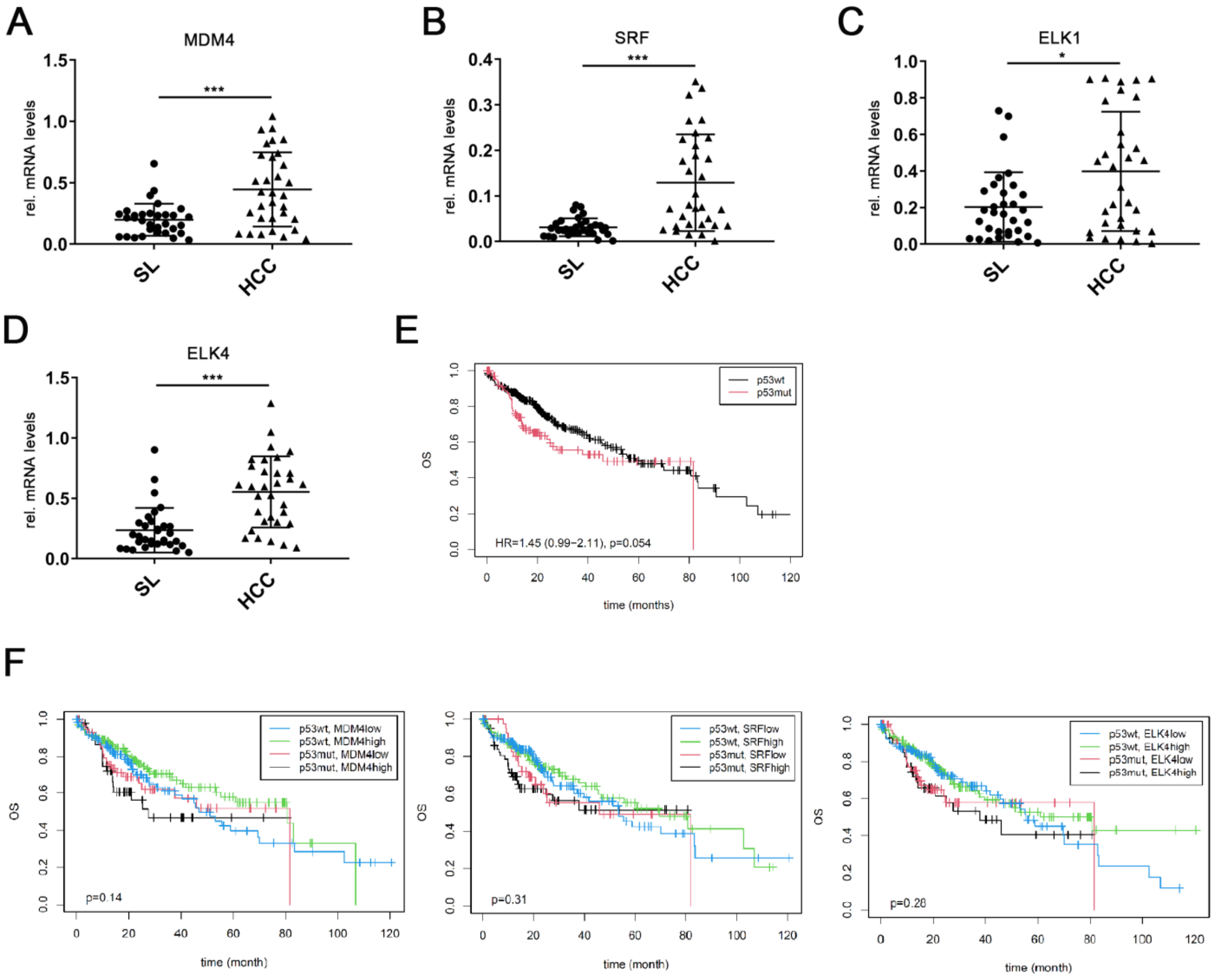

Figure S2. *MDM4* is upregulated in human HCC. (**A**) *MDM4* mRNA levels were increased in a second cohort of human HCCs compared to their corresponding non-tumor surrounding liver tissues ($n = 32$). Similar results were observed for (**B**) *SRF*, (**C**) *ELK1* and (**D**) *ELK4* in these samples. (**E**) Overall survival analysis revealed no difference between *p53* wildtype and *p53* mutated human HCC groups in the TCGA data set. (**F**) Similar results were observed after stratification in *p53* wildtype respectively mutated cases with low and high expression of *MDM4*, *SRF*, and *ELK4*. in the LIHC cohort. Abbreviations: SL, non-tumor surrounding liver tissues; HCC, hepatocellular carcinoma specimens; OS, overall survival. Mann-Whitney U test (panel **A**–**D**) and Log-rank test (**E**–**F**): * $p < 0.05$; *** $p < 0.001$.

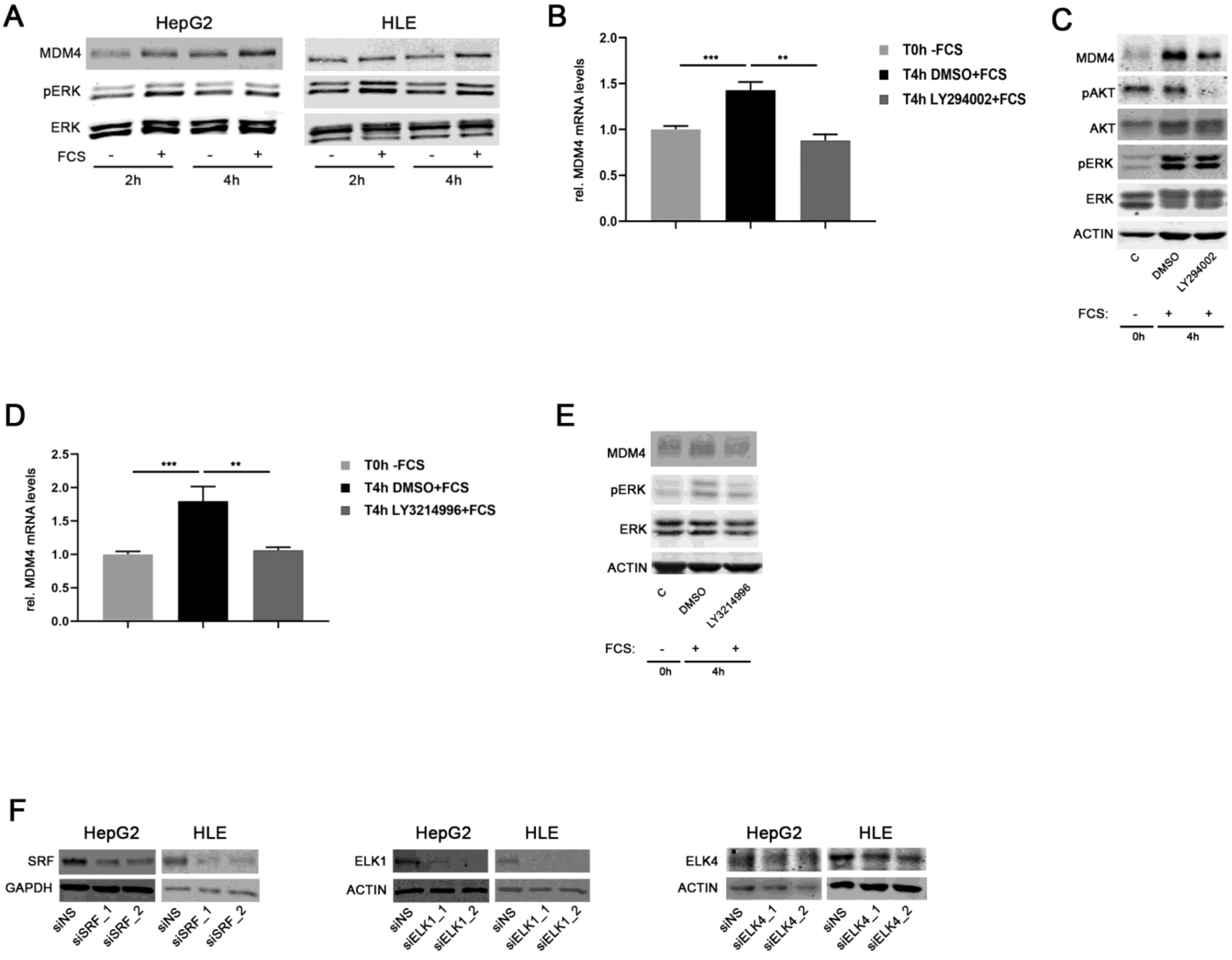

Figure S3. MDM4 protein induction upon FCS stimulation. (**A**) Increased MDM4 protein levels were observed in HepG2 and HLE 4h following FCS stimulation compared to starved control cells. (**B**–**C**) The PI3K inhibitor LY294002 rescued FCS-induced upregulation of *MDM4* mRNA and protein levels in serum-starved HepG2 cells Efficient inhibition of PI3K pathway was demonstrated by reduced AKT phosphorylation compared to untreated cells. Phosphorylation of ERK confirms the efficacy of FCS stimulation in comparison to overnight starved cells. (**D**–**E**) The ERK inhibitor LY3214996 rescued FCS-induced upregulation of MDM4 mRNA and protein levels in serum-starved HepG2. (**F**) Representative Western blots showing reduced expression of SRF, ELK1, and ELK4 proteins upon inhibition using gene-specific siRNAs in cell used for the luciferase assays. Kruskal-Wallis followed by Dunn´s test: ** $p < 0.01$, *** $p < 0.0001$.

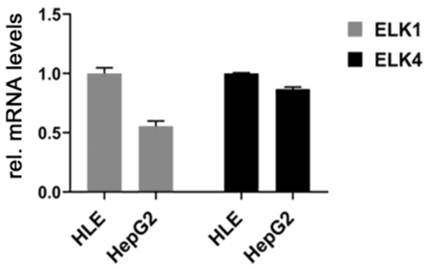

Figure S4. *ELK1* and *ELK4* expression levels in HCC cell lines. *ELK4* expression levels were similar between HepG2 and HLE cells, while the *ELK1* mRNA expression was lower in HepG2 cells compared to HLE cells.

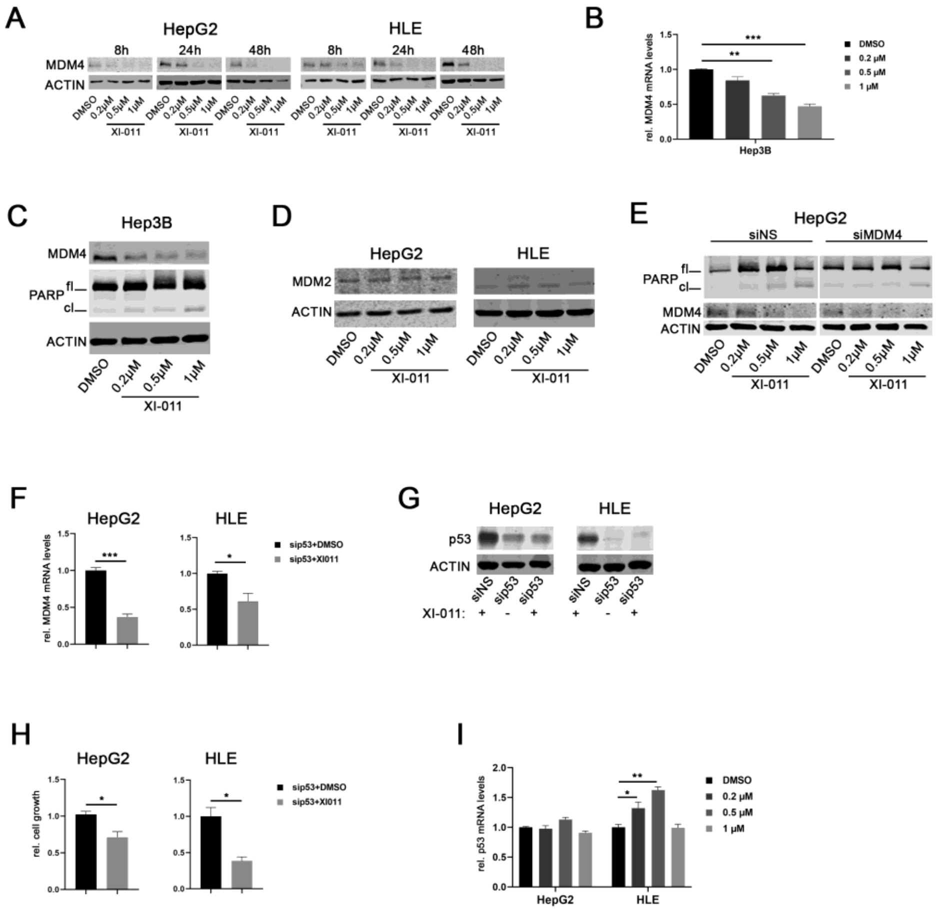

Figure S5. XI-011 induced biological effects are mediated by altered MDM4 levels independent from the *p53* gene status. (**A**) MDM4 protein levels in HepG2 and HLE cells at different time points following XI011 treatment as indicated. (**B**–**C**) Downregulation of *MDM4* mRNA and protein levels and induction of cleaved PARP protein was observed in Hep3B cells treated for 16 h with XI-011. (**D**) Representative Western blots evidenced no significant changes of MDM2 protein levels in both HCC cell lines treated for 16h using XI-011 compared to DMSO-treated control cells. (**E**) siRNA-mediated knock-down of MDM4 reduced PARP cleavage following XI-011 treatment in HepG2 cells compared to siNS-tranfected controls. (**F**) XI-011 treatment leads to downregulation of *MDM4* mRNA following siRNA-mediated knockdown of p53 expression in HepG2 and HLE cells. (**G**) Representative Western blotting images demonstrate efficacy of p53 knock-down in comparison to siNS transfected cells. (**H**) Cell growth was significantly diminished following p53 siRNA transfection combined with XI-011treatment when normalized to control cells. (**I**) Relative *p53* mRNA expression following XI-011 treatment using the indicated doses in HepG2 and HLE cells. Mann-Whitney U test (panel **F** and **H**) or Kruskal-Wallis followed by Dunn´s test (panel **B** and **I**) were used: * $p < 0.05$, ** $p < 0.01$, *** $p < 0.001$. Abbreviations: siNS, scrambled, nonsense siRNA; siMDM4, siRNA specifically targeting MDM4; sip53, siRNA specifically targeting p53; fl, full-length PARP protein; cl, cleaved PARP protein.

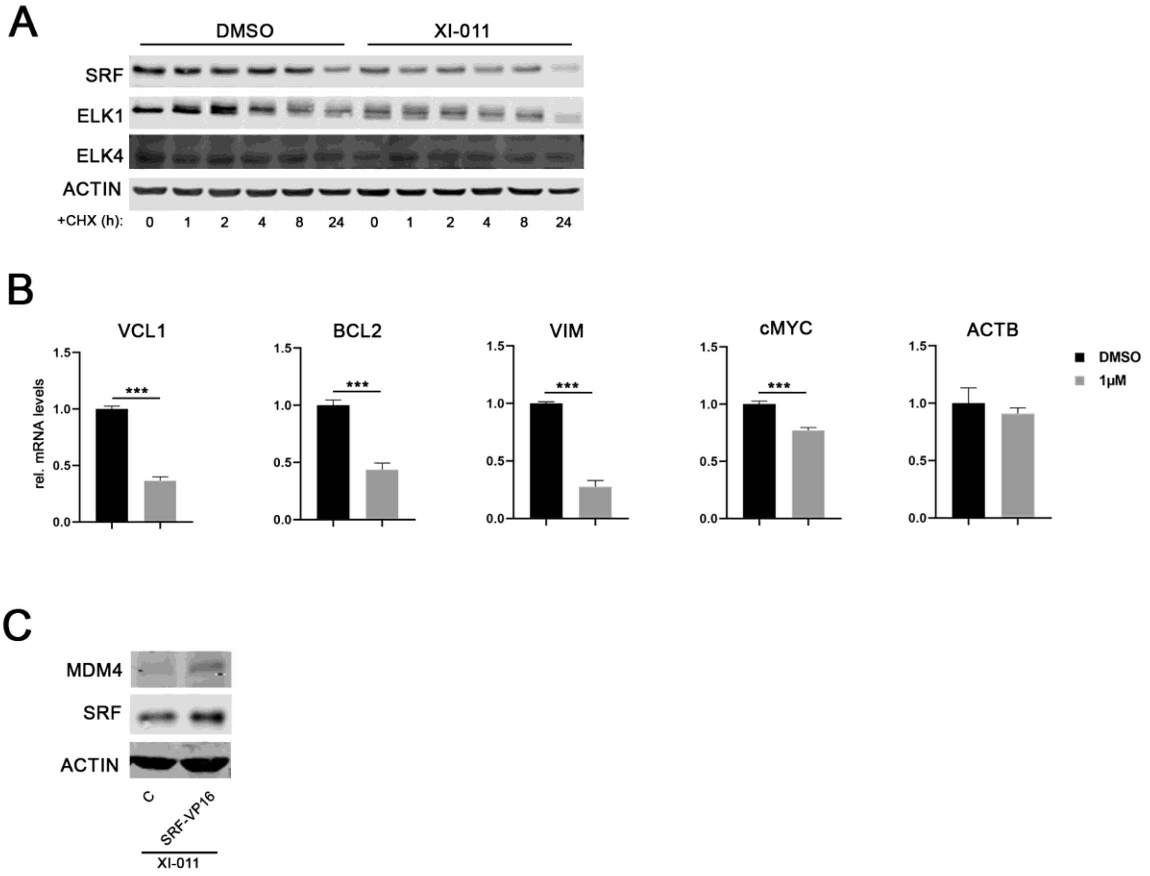

Figure S6. The half-life time of TF regulating *MDM4* transcription is not affected by XI-011 treatment in HCC cells. (**A**) The transcription factor (TF) protein half-life time was not altered following combined XI-011 (1 µM) cycloheximide (CHX) treatment in HLE compared to control cells. (**B**) XI-011 treatment results in reduced expression of most SRF target genes. (**C**) Overexpression of SRF prevented reduction of MDM4 protein following XI-011 treatment in HepG2 compared to control cells. Mann-Whitney U test was used: *** $p < 0.001$.

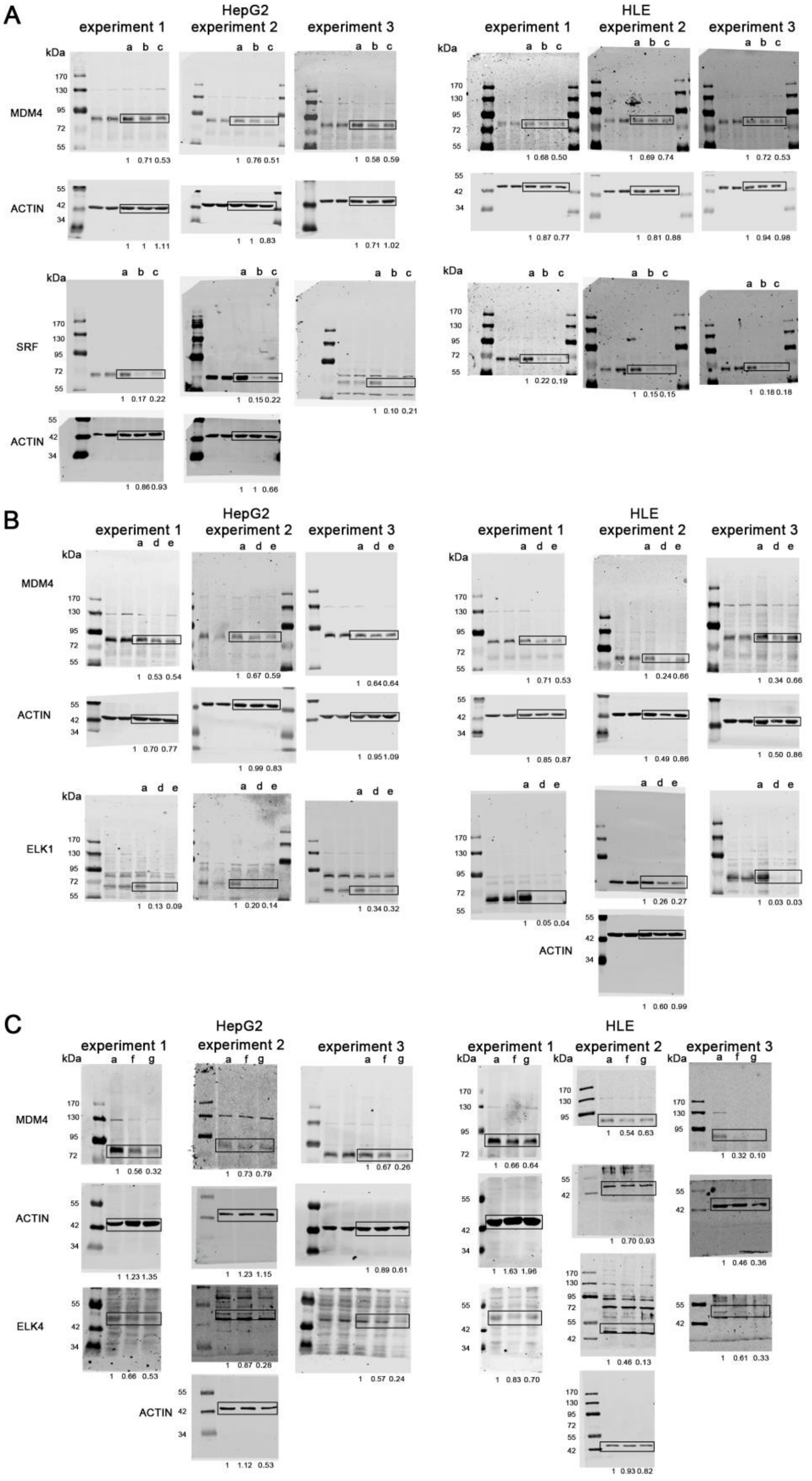

Figure S7. Western blotting image source. (**A**–**C**) Original Western blotting images displayed in Figure 2C,E,G and included in the corresponding protein level quantification histograms. Relative signal quantification is indicated below each corresponding band after normalization with control sample (a). Experiment 1 images are displayed in the main figure. Abbreviations: a, siNS scrambled, nonsense; b and c, siSRF_1 and _2, siRNA 1 and 2 specifically targeting SRF; d and e, siELK1_1 and _2, siRNA 1 and 2 specifically targeting ELK1; f and g, siELK4_1 and _2, siRNA 1 and 2 specifically targeting ELK4.

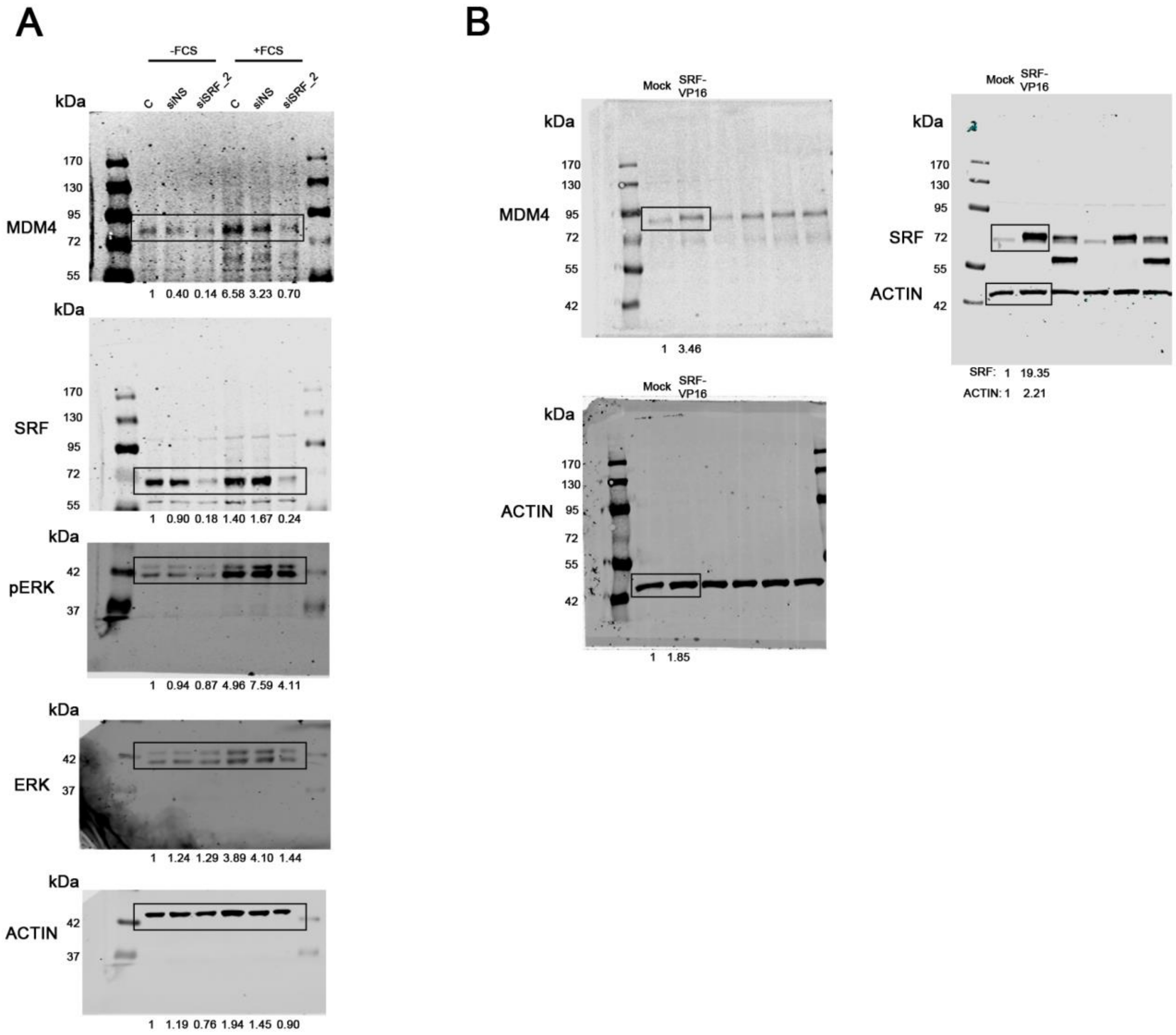

Figure S8. Western blotting image source. (**A**–**C**) Original Western blotting images included in Figure 2H and I. Densitometric quantification is indicated below each corresponding band after comparison with control sample.

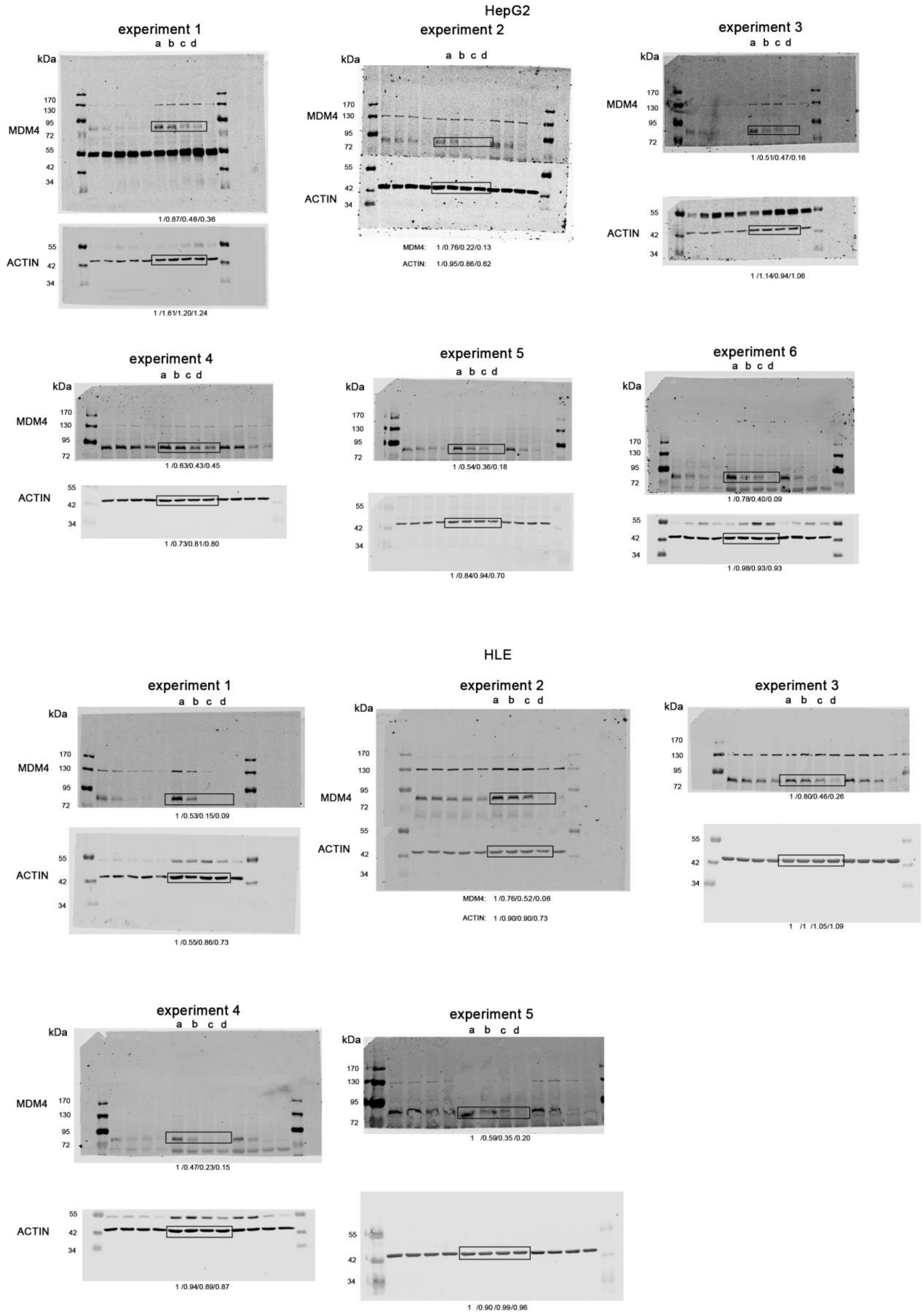

Figure S9. Western blotting image source. Original Western blotting images displayed in Figure 6B and included in the histogram describing MDM4 protein levels. Densitometric quantification is indicated below each corresponding band after comparison with DMSO control (a). Experiment 1 images are displayed in the main figure. Abbreviations: a, DMSO; b, 0.2 μM XI-011; c, 0.5 μM XI-011; d, 1 μM XI-011.

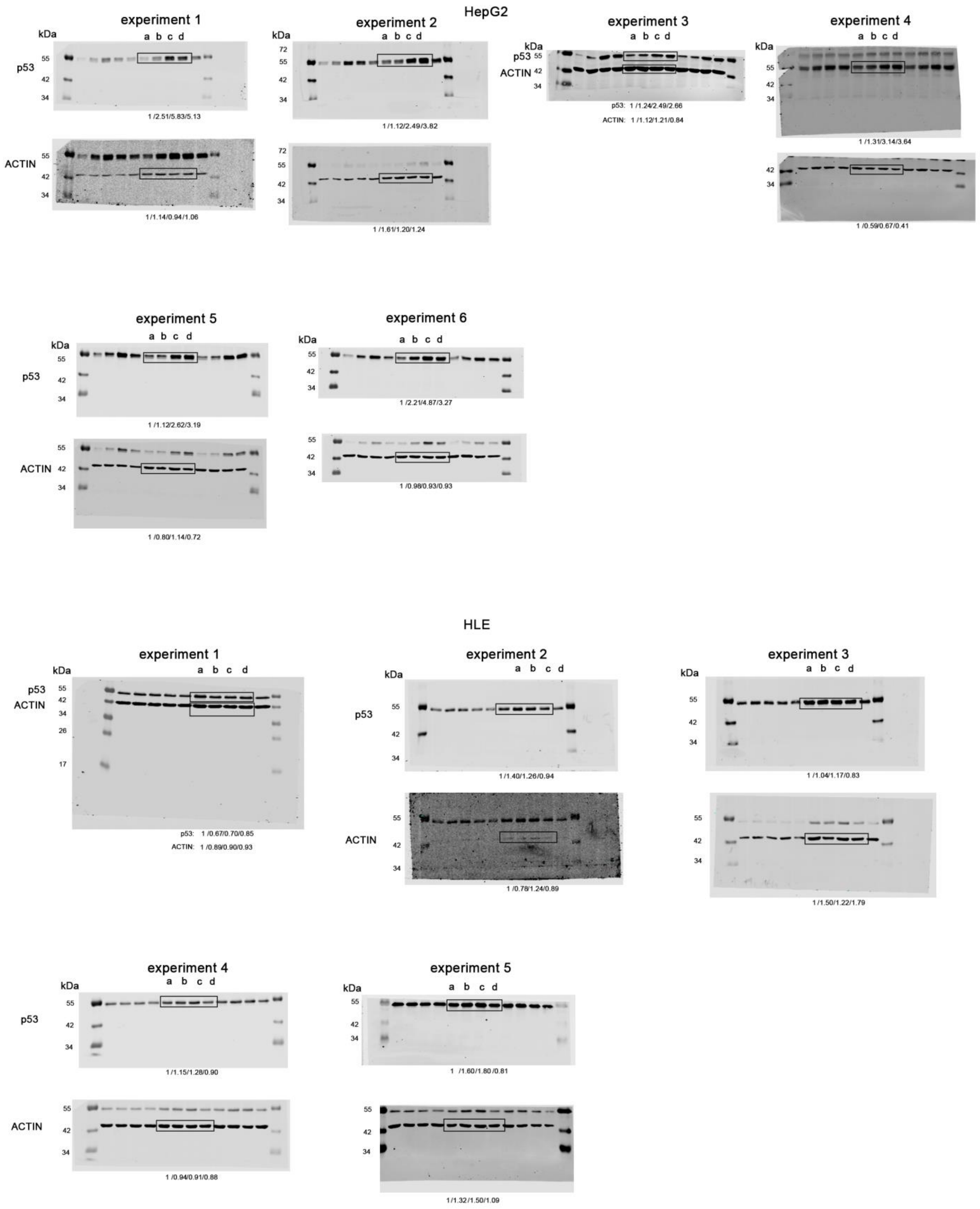

Figure S10. Western blotting image source. Original Western blotting images displayed in Figure 6D and included in the p53 protein level histogram. Densitometric quantification is indicated below each corresponding band after comparison with DMSO control (a). Experiment 1 images are displayed in the main figure. Abbreviations: a, DMSO; b, 0.2 μM XI-011; c, 0.5 μM XI-011; d, 1 μM XI-011.

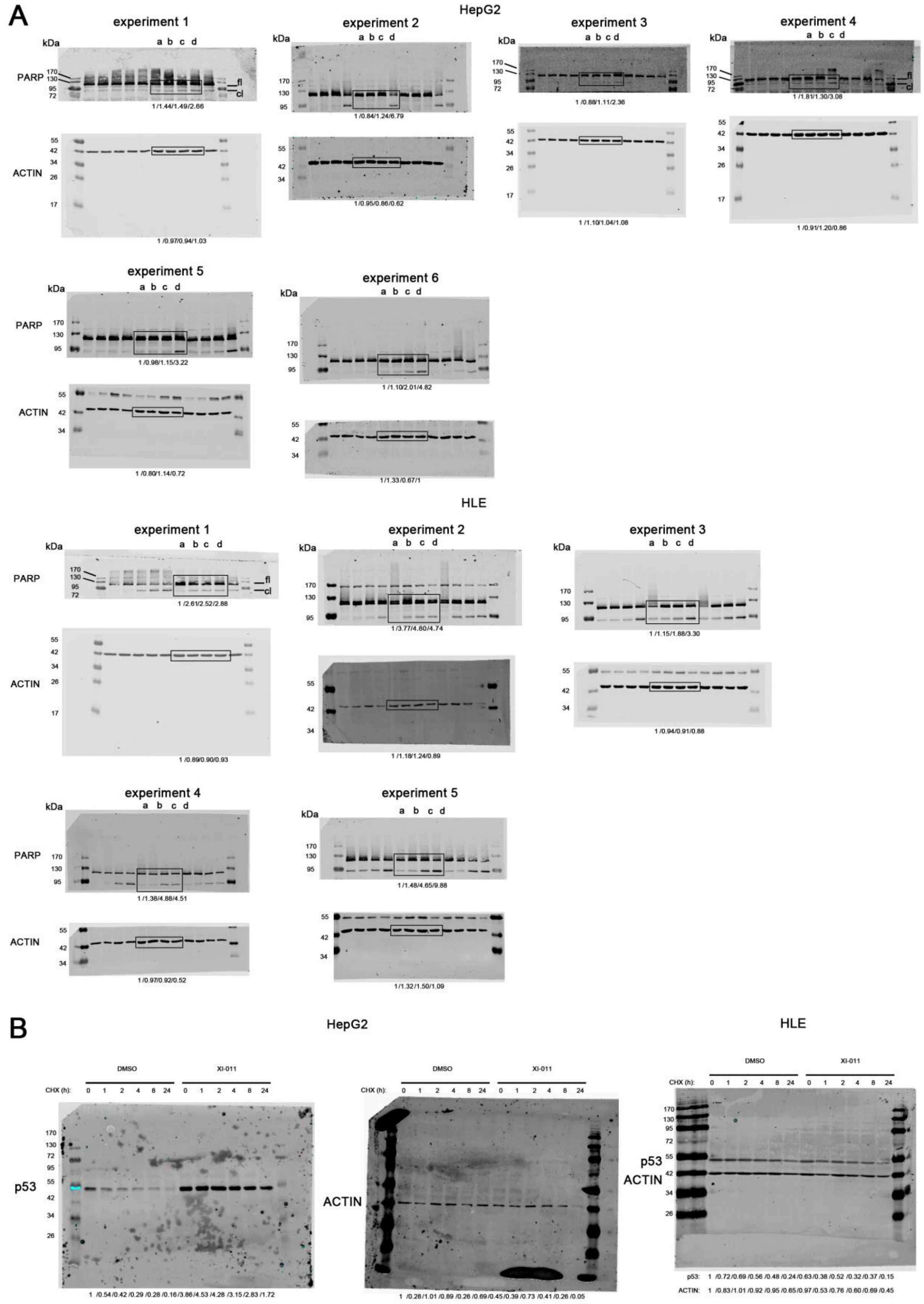

Figure S11. Western blotting image source. (**A**) Original Western blotting images displayed in Figure 6D and included in the PARP cleaved protein level graph. (**B**) Whole blot images shown in Figure 6F. Cleaved PARP densitometric quantification is indicated below each corresponding band after comparison with DMSO control (a) in panel A) or lane 1 in panel B). Experiment 1 images are displayed in the main figure. Abbreviations: a, DMSO; b, 0.2 μM XI-011; c, 0.5 μM XI-011; d, 1 μM XI-011; CHX, cycloheximide.

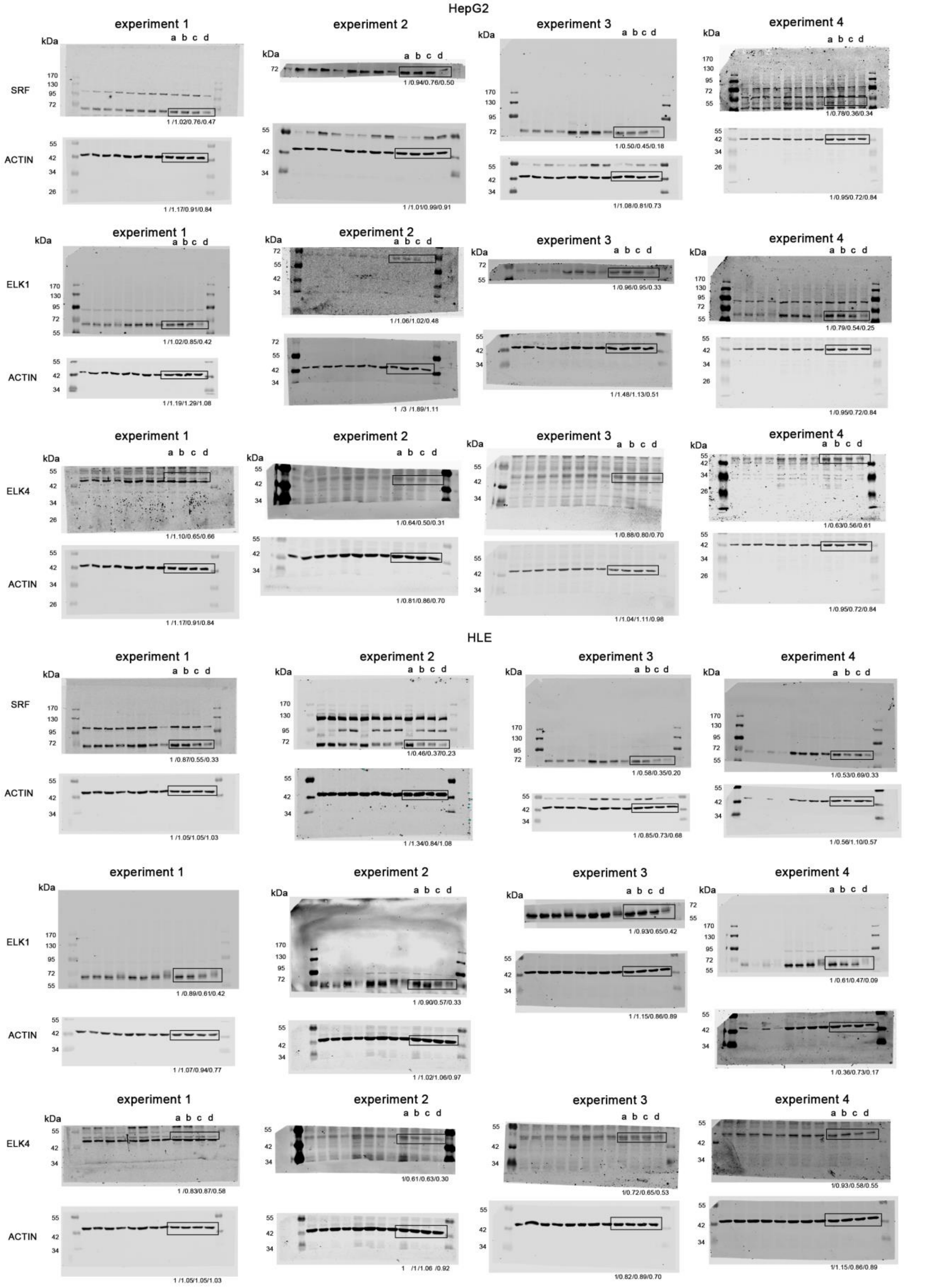

Figure S12. Western blotting image source. Original Western blotting images displayed in Figure 6H and included in the protein level graphs. Densitometric quantification is indicated below each corresponding band after comparison with DMSO control (a). Experiment 1 images are displayed in the main figure. Abbreviations: a, DMSO; b, 0.2 μM XI-011; c, 0.5 μM XI-011; d, 1 μM XI-011.

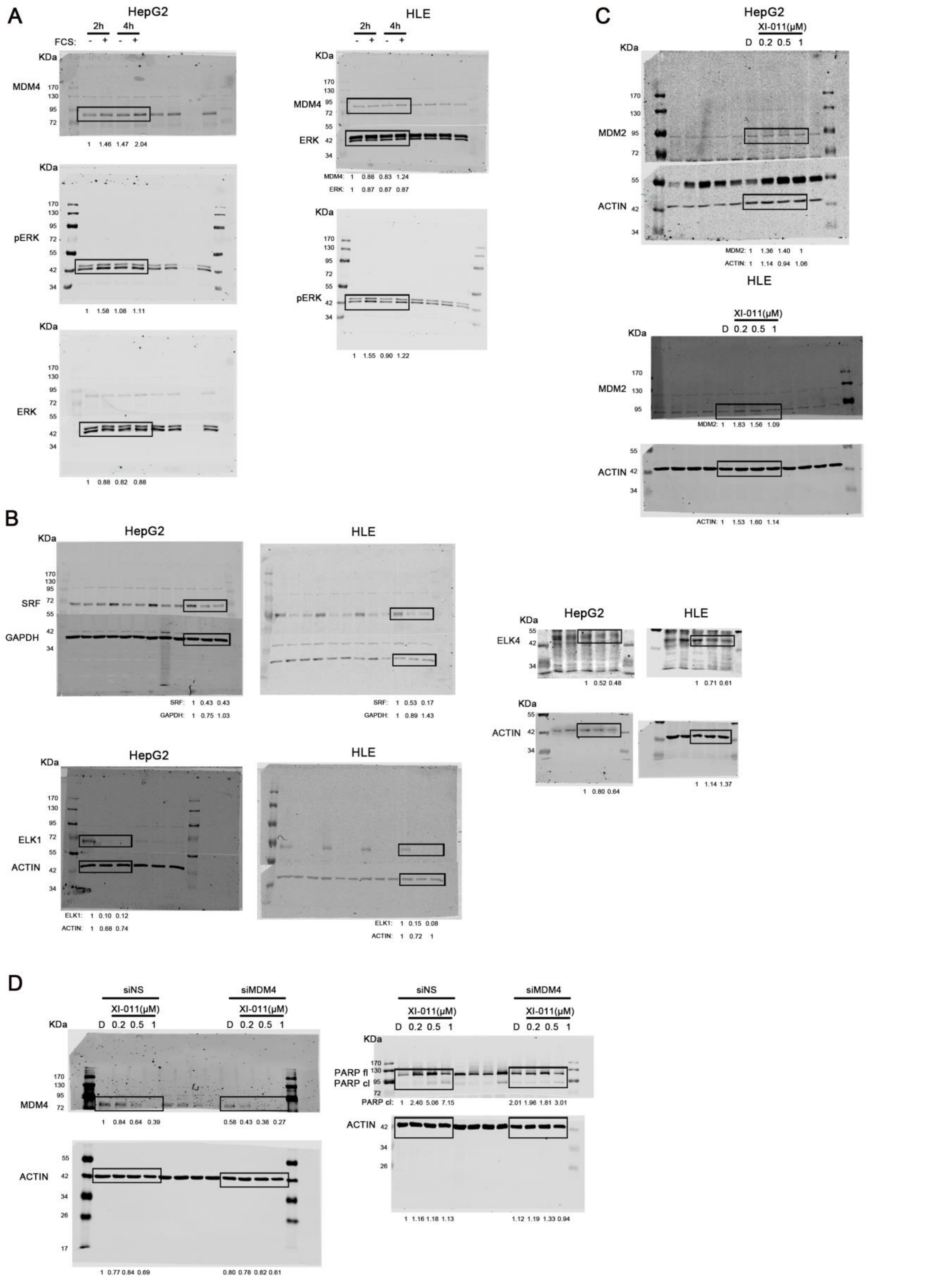

Figure S13. Western blotting image source. (**A**) Original Western blots included in. Figure S3A. (**B**) Original Western blots shown in Figure S3F. (**C**) Original Western blots shown in Figure S5D. (**D**) Original Western blots shown in Figure S5E. Densitometric quantification is indicated below each corresponding band after comparison with the first lane included in the indicated black boxes. Abbreviations: D, DMSO.

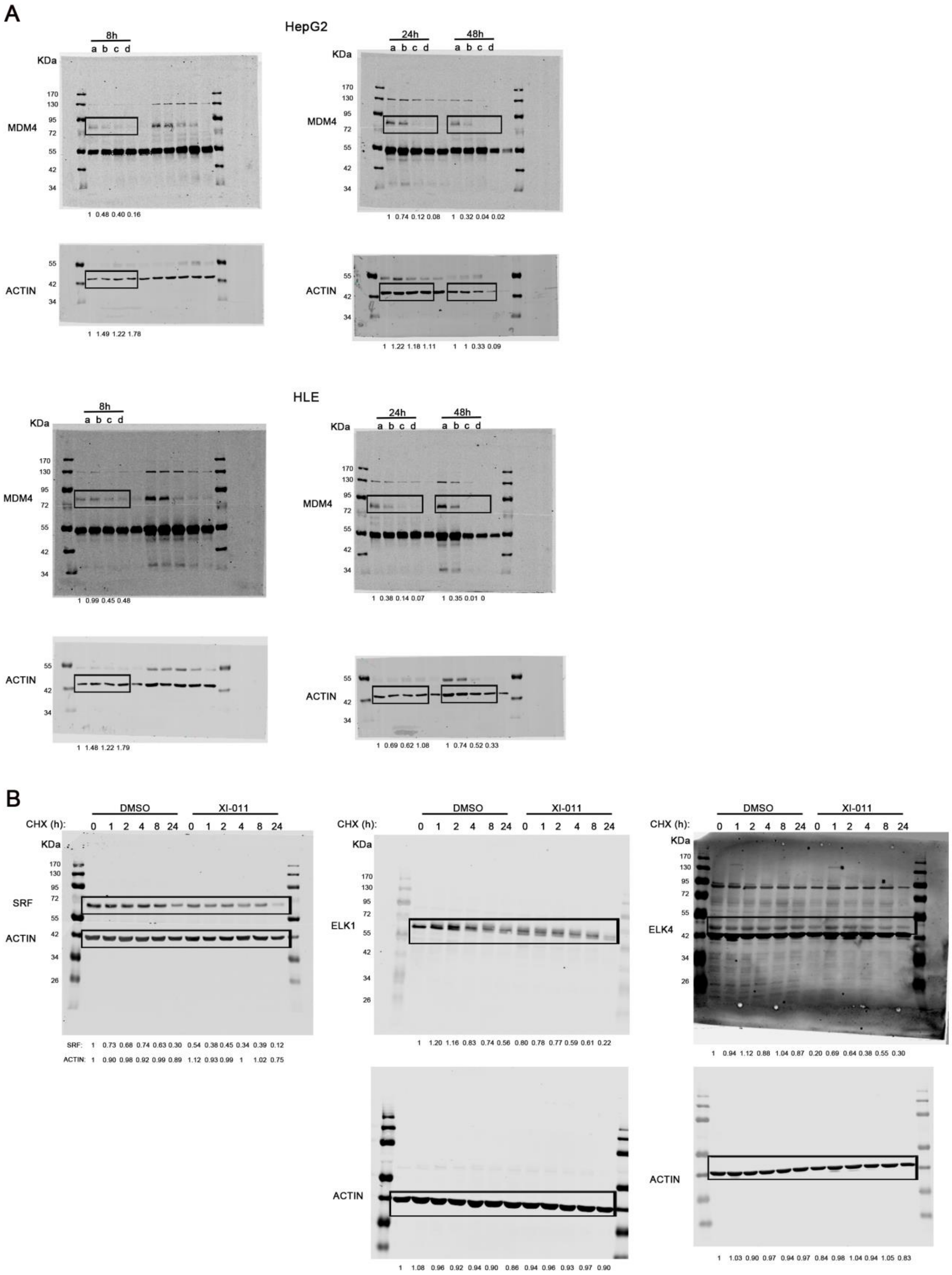

Figure S14. Western blotting image source. (**A**) Original Western blots included in Figure S5A. (**B**) Original Western blots shown in Figure S6A. Densitometric quantification is indicated below each corresponding band following comparison with DMSO control (a). Abbreviations: a, DMSO; b, 0.2 μM XI-011; c, 0.5 μM XI-011; d, 1 μM XI-011; CHX, cycloheximide.

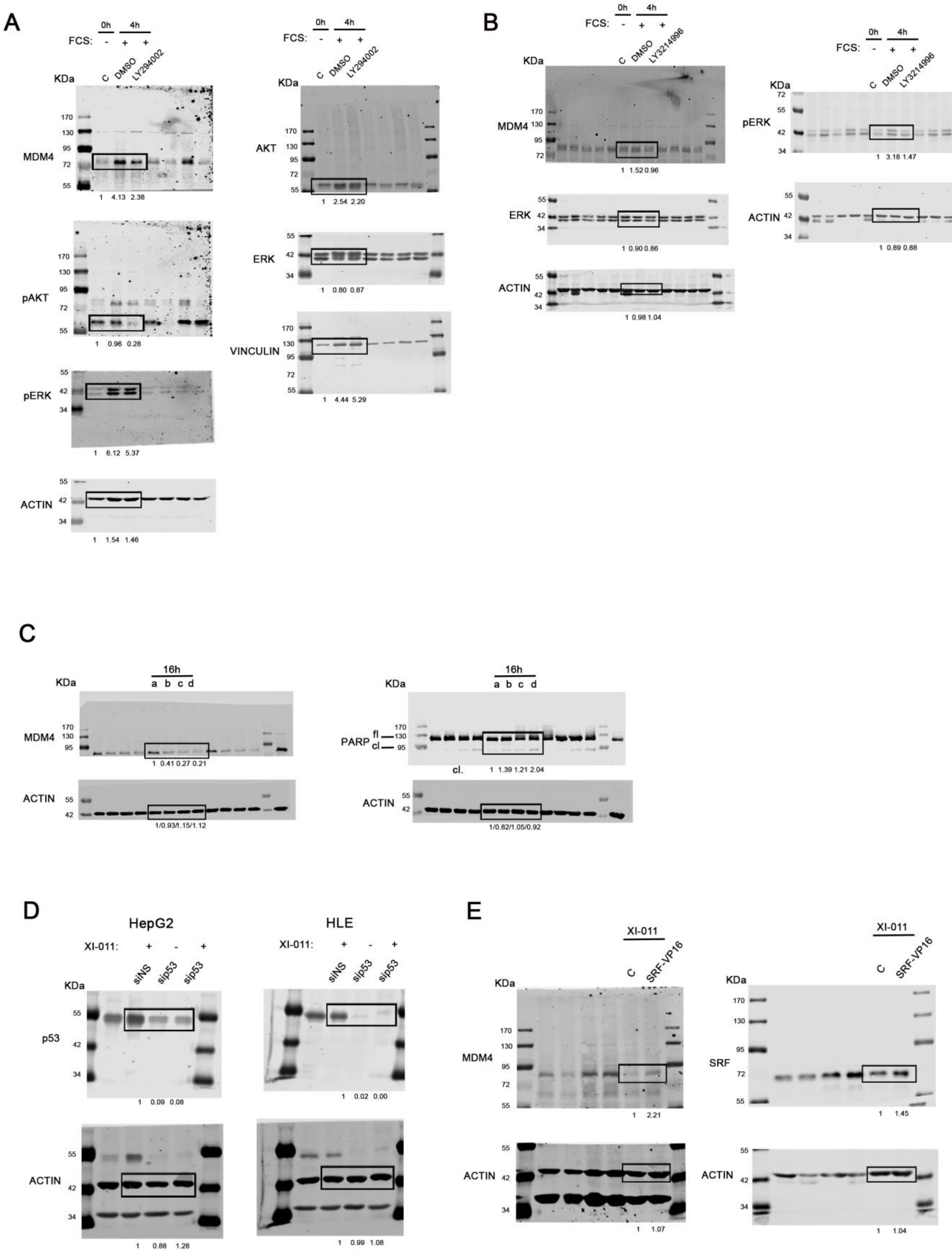

Figure S15. Western blotting image source. (**A**–**B**) Western blots displayed in Figure S3C. and E. (**C**–**D**) Western blots shown in Figure S5C and G. (**E**) Original Western blots included in Figure S6C. Abbreviations: a, DMSO; b, 0.2 μM XI-011; c, 0.5 μM XI-011; d, 1 μM XI-011; siNS, scrambled, nonsense siRNA; sip53, siRNA specifically targeting p53; fl, full-length PARP protein; cl, cleaved PARP protein.

Table S1. Clinicopathological features of HCC patients (for Figure.1A,C).

Variables	Features
**Gender**	
male	27 (73%)
female	10 (27%)
**Median age** (range)	57 (16–78)
**Etiology**	
HBV	8 (22%)
HCV	9 (24%)
alcohol	6 (16%)
cryptogenic	11 (30%)
others	3 (8%)
**Grading**	
well differentiated HCC	5 (13%)
moderately differentiated HCC	28 (76%)
poorly differentiated HCC	4 (11%)
**Tumor size**	
<2.0 cm	1 (3%)
2.0–5.0 cm	19 (56%)
>5.0 cm	14 (41%)
**UICC stage**	
I	25 (71%)
II	8 (23%)
III	0 (0%)
IV	2 (6%)
**Vascular invasion**	
present	10 (29%)
**Liver cirrhosis**	
present	18 (56%)

Table S2. Clinicopathological features of HCC patients (for Figure 1D, Figure S1/S2)

Variables Features	HCCB [a]	HCCP [b]
**No. of patients**	14	18
Male	11	12
Female	3	6
**Age** (Mean ± SD)	66.7 ± 7.9	66.5 ± 11.4
**Etiology**		
HBV	7	9
HCV	4	6
Ethanol	3	3
**Cirrhosis**		
+	11	13
−	3	5
**Tumor size**		
>5 cm	11	14
<5 cm	3	4
**Edmondson and Steiner grade**		
II	4	5
III	7	6
IV	3	7
**Alpha-fetoprotein secretion**		
>300 ng/mL of serum	6	5
<300 ng/mL of serum	8	13
**Survival after partial liver resection** (months, Means ± SD)	60.2 ± 20.4	15.5 ± 10.5

[a] HCCB, HCC with better prognosis/longer survival (survival >3 years following partial liver resection). [b] HCCP, HCC with poorer prognosis/shorter survival (survival <3 years following partial liver resection).

Table S3. List of the primary antibodies.

**ELK1**	**Mouse Monoclonal**	**COOH Terminus**	**Santa Cruz Biotechnology (Santa Cruz, CA, USA)**
pELK1 Ser383 (IHC)	Rabbit Polyclonal	region between residues 365–405	Abcam (Cambridge, MA, USA)
MDM4 (WB)	Rabbit Monoclonal	region between residues 125–175	Bethyl Laboratories (Montgomery, TX, USA)
MDM4 (IHC)	Rabbit Polyclonal	region between residues 125–175	Sigma-Aldrich (St. Louis, MO, USA)
ELK4 (SAP-1a)	Rabbit Polyclonal	Amino acids 154–320	Santa Cruz Biotechnology (Santa Cruz, CA, USA)
ELK4 (SAP-1a, IHC)	Rabbit Polyclonal	Amino acids 154–320	Novus Biologicals (Centennial, CO, USA)
SRF (WB)	Rat Monoclonal	Full length	Active Motif (La Hulpe, Belgium)
SRF (ChIP)	Rabbit Polyclonal	COOH terminus	Santa Cruz Biotechnology (Santa Cruz, CA, USA)
β-ACTIN	Mouse Monoclonal	NH2 terminus	Sigma-Aldrich (St. Louis, MO)
MDM2	Mouse Monoclonal	Amino acids 154–167	Santa Cruz Biotechnology (Santa Cruz, CA, USA)
p53	Mouse Monoclonal	Amino acids 11–25	Santa Cruz Biotechnology (Santa Cruz, CA, USA)
PARP	Rabbit Polyclonal	Caspase cleavage site	Cell Signaling (Danvers, MA)
GAPDH	Mouse Monoclonal	Full length	Bio-Rad Laboratories (Hercules, CA)
ERK	Rabbit Polyclonal	COOH terminus	Cell Signaling (Danvers, MA, USA)
pERK	Rabbit Polyclonal	Residues surrounding Thr202/Tyr204	Cell Signaling (Danvers, MA, USA)
AKT	Rabbit Polyclonal	COOH terminus	Cell Signaling (Danvers, MA, USA)
pAKT	Rabbit Monoclonal	Residues around Ser473	Cell Signaling (Danvers, MA, USA)
VINCULIN	Mouse Monoclonal	Full length	Sigma-Aldrich (St. Louis, MO, USA)

Table S4. Primer and siRNA sequence list.

MDM4-fw	5′-TCAGGTACGACCAAAACTGCC-3′
MDM4-rev	5′-AGTGCATGACCTCTTTAACAGTG-3′
SRF-fw	5′-GTCTCCTCTGGGGGCCTTA-3′
SRF-rev	5′-GCACTTGAATGGCCTGCAC-3′
ELK1-fw	5′-ACCCATCTGTGACGCTGTG-3′
ELK1-rev	5′-TCCCGTGAAGTCCAGGAGAT-3′
ELK4-fw	5′-AGCAGAGTTCATTTGGGAATGC-3′
ELK4-rev	5′-AACCAACAAATCTGTACCTGGC-3′
MDM4 (human HCC samples)	Hs00910358_s1 (TaqMan, Thermo Fischer Scientific)
SRF (human HCC samples)	Hs01065256_m1 (TaqMan, Thermo Fischer Scientific)
ELK1 (human HCC samples)	Hs00901847_m1 (TaqMan, Thermo Fischer Scientific)
ELK4 (human HCC samples)	Hs00360813_m1 (TaqMan, Thermo Fischer Scientific)
Mdm4-fw (murine samples)	5′-CTGTCTGGCTTCCTTTGTGG-3′
Mdm4-rev (murine samples)	5′-CTAAAGCGCTGCATCTCCTC -3′
Gapdh-fw (murine samples)	5′-TGGATCTGACGTGCCGC-3′
Gapdh-rev (murine samples)	5′-TGCCGTCTTCACCACCTT-3′
GAPDH-fw	5′-TGCACCAACTGCTTAGC-3′
GAPDH-rev	5′-GGCATGGACTGTGGTCATGAG-3′
VIM-fw	5′-GGACCAGCTAACCAACGACA-3′
VIM-rev	5′-TCCTCCTGCAATTTCTCCCG-3′
VCL1-fw	5′-CGATTACGAACCTGAGCTGC-3′
VCL1-rev	5′-GACCACTTGGTAGCTTCCCG-3′
ACTB-fw	5′-CCTCGCCTTTGCCGATCC-3′
ACTB-rev	5′-CGCGGCGATATCATCATCC-3′
BCL2-fw	5′-CCCGCGACTCCTGATTCATT-3′
BCL2-rev	5′-AGTCTACTTCCTCTGTGATGTTGT-3′
cMYC-fw	5′-TGGTGCTCCATGAGGAGACA-3′
cMYC-rev	5′-GTGATCCAGACTCTGACCTT-3′
p21-fw	5′-GGCGGCAGACCAGCATGACAGATT-3′
p21-rev	5′-GCAGGGGGCGGCCAGGGTAT-3′
MDM2-fw	5′-TCTGTGAGTGAGAACAGGTGTCAC-3′
MDM2-rev	5′-ACACACAGAGCCAGCCTTTC-3′
p53-fw	5′-TGACTGTACCACCATCCACT-3′
p53-rev	5′-CCATGCAGGAACTGTTACAC-3′
18s-fw	5′-AAACGGCTACCACATCCAAG-3′
18s-rev	5′-CCTCCAATGGATCCTCGTTA-3′
ElK1_ELK4 ChIP_2-fw	5′-CTCCATCTATGGTTTCCGGAGG-3′
ElK1_ELK4 ChIP_2-rev	5′-GCAGCATTTTGAAATGGCGAG-3′
SRF_ChIP_3-fw	5′-CTCCCCGGACTAGGATCTAC-3′
SRF_ChIP_3-rev	5′-AGGCCTCCGGAAACCATAGA-3′
upstream control ChIP-fw	5'-GCAGCTCATCAATCATTGCAC-3'
upstream control ChIP-rev	5'-CGGAAGCACCAATCTAGAGGA-3'
downstream control ChIP-fw	5'-ACCACAGTTTAAAAGCAACACCC-3'
downstream control ChIP-rev	5'-ACCAAGAGGTTCCAAGTATCTTT-3'
siELK1_1 (final concentration: 20nM)	5′-UGAAAUCGGAAGAGCUUAA-dTdT-3′
siELK1_2 (final concentration: 20nM)	5′-CGGAAGAGCUUAAUGUGGA-dTdT-3′
siSRF_1 (final concentration: 40nM)	5′-GUGAGACAGGCCAUGUGUA-dTdT-3′
siSRF_2 (final concentration: 40nM)	5′-UGAGUGCCACUGGCUUUGA-dTdT-3′
siELK4_1 (final concentration: 80nM)	5′-UUCUCCAUCUUCAGAAGAA-dTdT-3′
siELK4_2 (final concentration: 80nM)	VHS40843 (Thermo Fisher Scientific)
siMDM4 (final concentration: 40nM)	5´-AGCAACUAUACACCUAGAA-dTdT3´
sip53 (final concentration: 5nM)	5´-UGUUCCGAGAGCUGAAUGA-dTdT3´
nonsense siRNA (siNS)	5′-UUCUCCGAACGUGUCACGU-dTdT-3′
